# Challenges and Opportunities for Cervical Cancer Prevention Through HPV Vaccination in Ghana: A Public Health Policy Analysis

**DOI:** 10.1177/10732748251383280

**Published:** 2025-10-03

**Authors:** Eric Asempah, Ene Ikpebe, Michelle Wyndham-West, Mary E. Wiktorowicz

**Affiliations:** 1 Dahdaleh Institute for Global Health Research, 7991 York University, Toronto, ON, Canada; 2 Askew School of Public Administration and Policy, Florida State University, Tallahassee, FL, USA; 3 Design for Health and Inclusive Design Programs, 729842 Faculty of Design at OCAD University, Toronto, ON, Canada; 4 School of Health Policy and Management, Faculty of Health/Dahdaleh Institute for Global Health Research, 7991 York University, Toronto, ON, Canada

**Keywords:** cervical cancer, vaccination, priority setting, HPV, resource allocation, Ghana

## Abstract

**Introduction:**

Cervical cancer constitutes a critical public health challenge in Ghana, with high morbidity and mortality despite the global availability of effective prophylactic Human Papillomavirus (HPV) vaccines. This study examines the policy discourse surrounding the implementation of a nationwide HPV vaccination program in Ghana, analyzes stakeholders’ perspectives on programmatic promotion, and assesses the extent of institutional prioritization.

**Methods:**

Eight key informant interviews were thematically analyzed using NVivo; and a cross-sectional online survey of 215 participants was descriptively analyzed using SPSS.

**Results:**

Thematic analysis of interviews revealed core policy challenges: weak prioritization, inadequate resource allocation, and policy framings that lacked discourse on the right to health. Survey data demonstrated marked improvement in HPV awareness (76.6%) and substantial interest in vaccination (64.2%), suggesting a shifting public health landscape influenced by media engagement and growing health literacy.

**Conclusion:**

Findings underscore insufficient prioritization stalled the institutionalization of a national cervical cancer prevention strategy creating a critical implementation gap. However, the relatively late average age of sexual debut offers a strategic window for effective HPV vaccine delivery. Importantly, the convergence of increased public awareness, heightened receptivity to vaccination, and the availability of external funding mechanisms, such as support from Gavi, presents a timely and actionable opportunity for policy advancement. This study highlights the imperative for renewed governmental commitment to cervical cancer prevention, emphasizing the imperative to operationalize HPV vaccination as a core component of Ghana’s public health infrastructure.

## Introduction

Cervical cancer is the second most prevalent cancer among women between the ages of 15 and 44 in Ghana.^1^ According to recent global estimates, cervical cancer accounted for approximately 660 000 new cases and 350 000 deaths worldwide in 2022, with 88% to 94% of the burden occurring in low- and middle-income countries (LMICs).^2,3^ Incidence rates in LMICs are nearly double, and mortality rates are up to fivefold higher, compared with high-income countries (HICs).^2^ These disparities are largely driven by limited access to human papillomavirus (HPV) vaccination, organized screening programs, and timely treatment services.^2,3^ In contrast, HICs continue to report substantially lower incidence and mortality rates, in some regions as low as 5.6 to 6.2 per 100 000 women due to sustained policy interventions such as widespread HPV immunization and organized cervical cancer screening.^4,5^ Gradually, some sub-Saharan African countries like Rwanda, have begun to implement a comprehensive national HPV vaccination program targeting girls under 15 years of age, and have seen steady decline in cervical cancer incidence.^6-9^ A larger segment of LMICs, however, remain less successful in designing and implementing HPV vaccination program in pursuit of cervical cancer prevention.^10,11^ As a result, the disease persists disproportionately in LMICs. In Ghana for example, knowledge about HPV, cervical cancer, and HPV vaccination is low. A study of 285 adolescents in 2020 found that 91.2% were not familiar with HPV, and 95.4% had not heard of HPV vaccination^12^ ^(p. 1)^ Previous studies reported similar findings, thus indicating low public education on cervical cancer and its prevention and/or control within the country.^13-15^

This study addresses the following questions: (1) what issues have stakeholders focused on as they have sought to bring policy attention to the HPV vaccination program in Ghana? (2) What are current levels of public knowledge of the role of HPV vaccination in cervical cancer prevention and receptivity to vaccine uptake? The study aims to make valuable contributions to the ongoing efforts to establish a successful vaccination program in Ghana and identify opportunities to improve HPV vaccination strategies, informed by stakeholder perspectives and public awareness.

## Agenda Setting and HPV Vaccination in Ghana

This study draws on agenda setting as a heuristic underpinning within the policy process literature. Agenda setting is the process by which policy topics gain or lose the attention of policymakers.^16^ As a policy agenda refers not only to the list of problems government aims to solve but also their causes, solutions, and any results of actively deliberating on a specific collection of issues,^17,18^ agenda setting may also be defined as the intricate work of identifying and defining problems and justifying the use of scarce resources to address them, often to the neglect of other issues.^17^ A range of state and non-state actors are involved in the process. Interest groups, for instance, are single or multi-issue groups devoted to advancing the presence of their concerns as priorities for policymakers.^19,20^ Research think tanks can be critical academic or sometimes government-driven participants in the policy process who generate new knowledge and data based on which policy discussions governments prioritize.^21^ Similarly, corporations partake in lobbying related activities involving information and financial resources.^22^ Citizens and nonprofit organizations are also involved in agenda setting process in a variety of ways including membership and participation in community groups,^23^ individual participation using e-government systems^24,25^ or through social media use.^26,27^ Both government and non-government policy actors have been involved in agenda setting concerning HPV vaccination in Ghana, from global to local levels. For instance, the 2018 WHO strategy to end cervical cancer served as an incentive for state ministries of health, politicians, researchers, citizens, and the healthcare industry to concentrate efforts on the issue.^28^ Specifically, the stated goal of the strategy was for 90% of girls to be fully vaccinated with HPV vaccine by age 15 years. With this and other specific policy goals, the WHO strategy influences not only problem definition, but how solutions are crafted. Similarly, the Ghanaian Ministry of Health (MOH) has a general cancer policy ie, the National Strategy for Cancer Control in Ghana (NSCC), though it has not yet developed a specific policy governing the prevention and control of cervical cancer. Reichenbach^29^ noted that cervical cancer falls off governmental policy priority and does not inspire strong policy advocacy because of the “social construction” of the disease as sexually transmitted. Even though the NSCC outlines an HPV vaccination strategy, the sexualized connotation of cervical cancer makes garnering the necessary attention and resources from the government more challenging. Although Ghana’s HPV vaccination rollout, initially planned for 2022 through school-based delivery and community outreach, has not yet materialized^30^ ^(p. 2)^, the WHO Ghana Country Office suggest that the country is scheduled to introduce the vaccine by the end of 2025.^31^ Cost-effectiveness analyses suggest that introducing the vaccine program would be more cost-effective than taking no action,^30^ aligning with evidence from similar analyses in other countries.^32-34^ The recent WHO guidance to add the *single-dose* HPV vaccine (Cecolin^®^),^35^ at a lower cost than 2- or 3-dose HPV vaccines,^36^ could foster agenda setting, especially for countries yet to incorporate an HPV vaccine program. In clarifying agenda setting issues like resource insufficiency and low government priority, this study addresses some immediate term challenges for Ghana to save on public health costs in the long term. The agenda setting literature frames the examination of policy actors’ participation in HPV vaccination policy discussion in Ghana, acknowledging that they may not be equally aware of, or interested in, advancing an HPV vaccination program. Similarly, our estimates of public awareness of the risk of cervical cancer and of role of HPV vaccination in public protection is captured within agenda setting as public opinion offers an incentive for public officials to commit more attention to vaccination programs.

## Methods and Materials

Data were collected using a mixed-methods approach, combining qualitative semi-structured interviews and quantitative surveys. Key informant interviews (KIIs) elicit policy and system-level barriers and delivery insights from those shaping or executing HPV vaccination, while survey questions administered quantifies knowledge, attitudes, and intentions in the general population with acceptable precision. This pairing is consistent with Ghana-based HPV studies that combine qualitative insights with quantitative measures of awareness and acceptance in communities.^37,38^ Key informants identified were stakeholders with interest in cervical cancer prevention and control and/or those who were involved in public health policymaking in Ghana. Applying a snowball search, a list of potential stakeholders was emailed to seek participation, and to suggest other stakeholders they deemed important informants for the study. Interview guide was designed to obtain key informant perspectives on the policymaking process concerning HPV vaccination in Ghana (Supplemental File 1). All interview participants consented to participate in the study, understood their role before participating, were anonymized, and identity coded. Interview responses were coded and analyzed using NVivo 12 for iterative category organization, and theme identification. Codes were refined to establish clear themes by removing duplicates and merging sub-categories. Themes generated from interview data analyses were triangulated through survey data results.^39,40^ Data coding was by EA and verified by MEW. The survey instrument was developed using Google Forms, and the link was disseminated electronically via email, phone, word-of-mouth contacts, and WhatsApp group pages. No socioeconomic and educational barriers were set allowing the survey to collect diverse opinion. Inclusion criteria for the survey was respondent’s ability to read and understand questions and residency in Ghana. Informed consent statement appeared on the first page of the Google Forms, and participants were required to indicate agreement before proceeding. The survey was designed to gauge current public knowledge of HPV and receptivity to vaccine uptake, updating previous studies.^41,42^ Demographic information (gender, age, education, and employment) was used as independent variables in descriptive statistics including correlations. To evaluate the level of overall risk of cervical cancer, respondents were asked their age at first sexual experience. Questions were asked about awareness of HPV, where information was attained, knowledge of HPV infection transmission through sex, and knowledge of HPV genotypes causing cervical cancer, and whether respondents were aware of a vaccine and of a nationwide HPV vaccination program. With vaccine hesitancy on the rise, participants were asked if they had taken the HPV vaccine, or whether they intended to take it in the future should it be introduced by the government. To evaluate their views on government resource allocation to health, respondents were asked whether they think the government has the resources to conduct nationwide HPV vaccination for those who need it, whether the government is committed to public education on cervical cancer, and whether women’s health was a governmental priority. Respondents’ knowledge of cervical cancer policy and government priority setting was evaluated by asking about their knowledge of a policy on cervical cancer prevention and control and the government’s commitment to prioritizing cervical cancer prevention.

The study methodology followed the COREQ 32-item checklist for qualitative research^43^ and the STROBE guidelines for observational studies.^44^

Ethical approval was obtained on November 12, 2021, from the Human Participants Review Sub-Committee, York University’s Ethics Review Board, Office of Research Ethics (Certificate #: STU 2021-137). Key information interviews were conducted from January 5^th^ 2022 to April 5^th^ 2022 while survey data collection started on December 21^st^ 2021 and ended on March 15^th^ 2022.

## Results

### Interviews

Eight interviews were conducted overall; two interviews were conducted in person. One interview conducted via Zoom, was audio and video recorded with the informant’s permission that was transcribed using rev.com, crosschecked for omissions, transcription errors and manually corrected. Five anonymized key informants completed the interview questions and returned their responses by email. Empirical studies show that core themes in focused, relatively homogeneous domains typically stabilize within 6-12 interviews; we monitored for saturation and observed no novel codes after interview 7, supporting adequacy at n = 8.^45,46^ The analysis uncovered six themes: awareness, media influence in vaccine uptake, policymaking, priority setting, resource allocation, and right to health that were further reduced to 4 main themes based on commonalities (Supplemental File 2). Despite an emphasis on media influence in vaccine uptake, it is considered a sub-category of the “awareness” theme. Media influence serves a crucial tool for raising awareness of cervical cancer. The sub-themes of priority-setting and resource allocation are merged into 1 theme due to their direct link to government authority to allocate resources using state apparatus. The right to health theme reflects individuals’ ability to access resources to protect their health.

### Survey

The survey received 215 responses. Of these, 14 were excluded from respondents who resided outside of Ghana. A total of 201 survey responses were deemed valid, with 64 (31.8%) from male and 137 (68.2%) from female respondents, and the data were statistically analyzed using IBM Statistical software for Social Sciences (SPSS) (version 28.0). With n = 201, a conservative single-proportion precision calculation at 95% confidence (Cochran formula with P = .50) yields a half-width margin of error ≈ ±6.9%, which is acceptable for descriptive estimates of HPV vaccine awareness, attitudes, and intentions.^47,48^ Some respondents provided incomplete answers, which were treated as missing data and excluded during the data cleaning process. The data were categorized based on the questions asked and analyzed using Chi-square tests to test relationships between variables. Relationship measures were interpreted using Phi’s coefficient, and a P*-value* less than .05 was considered statistically significant (Supplemental File 3). Most respondents were over 26 years old (94.6%). Out of 198, 14 (7%) reported debuting sex before age 17. Although the link between debut age and HPV awareness was weak, 95 (69.3%) women and 44 (69%) men, debuted sex after 17 years (Table 1). The analysis triangulates the six themes identified in the key informant interviews with the survey results.

**Table 1.** Sociodemographic Characteristics and Survey Responses

	Male	Female	Frequency	Percent
Gender				
Male	64	N/A	64	31.8
Female	N/A	137	137	68.2
Age range				
18-25	6	5	11	5.5
26-35	16	77	93	46.3
36- up	42	55	97	48.3
Education				
Junior high school	1	1	2	1.0
Senior high school	2	0	3	1.5
Vocational	1	1	2	1.0
University	53	111	164	81.6
Other	6	24	30	14.9
Age of debut sex				
Not yet	5	5	10	5.0
<17	4	10	14	7.0
>17-26	30	59	89	44.3
27+	14	36	50	24.9
Prefer not to answer	11	24	35	17.4
Are you aware or have you heard of HPV?				
Yes	38	125	163	81.1
No	26	11	37	18.4
If you have answered YES, how did you hear about HPV?				
Self reading	11	21	32	15.9
National education program	3	16	19	9.5
Government owned TV station	1	2	2	1.0
Privately owned TV station		1	1	0.5
Government owned radio station		1	1	0.5
Privately owned radio station	9	17	26	12.9
Internet	8	19	27	13.4
School	1	47	57	28.4
Do you know whether HPV infection can be spread through sex or not?				
Yes	38	116	154	76.6
No	24	15	39	19.4
Maybe	2	6	8	4.0
If your country has introduced the HPV vaccine, have you taken the shot yet?				
Yes	1	13	14	7.0
No	49	105	154	76.6
If you have not taken the HPV vaccine yet, will you be willing to take the shot when your country introduces it?				
Yes	26	103	129	64.2
No	10	9	19	9.5
Maybe	21	13	34	16.9
Do you think the government will introduce HPV vaccine at some point?				
Yes	15	48	63	31.3
No	5	9	14	7.0
Maybe	38	56	94	46.8
Do you think the government has the resource to conduct a national HPV vaccination for those who need it?				
Yes	29	54	83	41.3
No	14	42	56	27.9
Maybe	21	41	62	30.8
Is the government effort to educate the public on HPV related cervical cancer adequate?				
Yes	6	20	26	12.9
No	43	100	143	71.1
Maybe	17	14	31	15.4
Are you aware if your country has any specific policy on cervical cancer prevention and control?				
Yes	18	57	75	37.3
No	46	78	124	61.7
Is the government committed to prioritizing cervical cancer prevention in your country?				
Yes	31	47	49	24.4
No	15	45	92	45.8
Maybe	18	45	59	29.4
Do you think women’s health is a priority to the government?				
Yes	31	47	78	38.8
No	15	45	60	29.9
Maybe	18	45	63	31.3
Are you aware if your country has any specific policy on cervical cancer prevention and control?				
Yes	18	57	75	37.3
No	46	78	124	61.7
Have you ever demanded your right to heath at any time in your life in your country?				
Yes	29	65	91	45.3
No	35	73	108	53.7

### Awareness of HPV

The interviews suggest a lack of public awareness of HPV and cervical cancer. Key informant (GH-001-PHY) claimed that “…The health workers themselves are not even aware about it [ie, *HPV and cervical cancer*] and are not much informed about it, so how much more [ie*, health workers*] giving the information out. So, there’s a deficit there…”

This deficit in awareness notwithstanding, it is crucial to note that an increasing number of Ghanaians are becoming aware of HPV infections and cervical cancer. This is important for overall cervical cancer awareness, as evident in our findings compared with existing literature. Still, stakeholders continue to encourage further information dissemination. According to a key informant from a women’s advocacy group,

“…information [ie, *on HPV and cervical cancer*] that is released by official sources, such as the Ghana Health Service [GHS], should be promoted on news websites and mass media channels, such as radio, newspapers, and TV, as well as by using Facebook ads, which guarantees wide reach and engagement” (GH-001WGP).

The same key informant further noted that;

“… in order to educate women, people with specific religious (eg, religious leaders) or political beliefs, and people living in urban communities, with the goal to increase knowledge and trust in vaccines, targeted programs such as community outreach and media campaigns [*must be developed*]” (GH-001WGP).

Eighty-one percent of survey respondents are aware of HPV, learning about it through various sources, especially schools. However, most respondents (71.1%) were of the view that government effort to educate the public on cervical cancer is inadequate. The relationship between respondent interest in government commitment to cervical cancer prevention and public education is strong (*Phi (φc) = 0.515*). At the same time, correlation of education with other variables, including willingness to vaccinate, awareness of policy on cervical cancer prevention, government resource allocation, and right to health yielded statistically insignificant outcomes (P*-value >.05*).

### Policymaking

To emphasize the need to address cervical cancer, a physician key informant stressed that, “Cervical cancer and HPV vaccination should be prioritised just as breast cancer is done in Ghana” (GH-002-PHY). The view of the key informant was that Ghana is not deficient in policymaking; however, the problem lies in implementation of the stated policies,

“We [ie, *Ghana*] have all the good policies, like the cancer control policy in 2010, excellent policy! The problem is implementing it. And it has always been put on the lack of funds. So, I wouldn't say we lack the policies. For the policies we have about the best you can think about. We have a very good cancer control policy for over a decade, but they’ve not been implemented. We know who to screen, we know who to vaccinate. They are all in the policy, but it has never been implemented, and we blame it on the lack of funds. So, I wouldn’t say that we don’t have the capacity to make the policy. The problem is the implementation; that has been a problem” (GH-002-PHY).

In similar response to the role of policymakers and their influence in HPV vaccination in Ghana, a Women’s advocacy group key informant, explained that,

“The Ghana Health Service (GHS) and some health-related civil society organizations have the power to influence HPV vaccination in Ghana. The development of a “National Strategy for Cancer Control and prevention policy” by the GHS and ability to influence public education in Ghana, is a great stride.” (GH-001-WGP)

The same key informant however asserted that, “much is expected to be done [*by the government*]” (GH-001WGP). Aligned with her remark, one hundred and twenty-nine (64.2%) respondents are willing to take the HPV vaccine when it is introduced in Ghana. Premised on the survey response, it is hypothesized that introducing the vaccine to the target population promises significant uptake.

### Priority Setting and Resource Allocation

A physician key informant asserted that when it comes to government attention to cervical cancer prevention, “[T]here is no effective cervical cancer prevention program in Ghana” and continued that,

“The policy on control of cervical cancer expired in 2016 [*referring to the NSCC*] and as of now, no functional policy. There is non-existent program on cervical cancer control.” (GH-003-PHY)

Another physician key informant noted that,

“We [*i.e., Ghana*] are getting around 3,000 cervical cancer cases diagnosed a year [and] about 1,500 around that are dying in a year. That is significant. If you put all together, we have a burden to deal with. It is necessary that we invest our energies [i]n this area [*referring to HPV vaccination*]” (GH-001-PHY).

The survey findings indicate varying perspectives on the government’s prioritization of women’s health, 78 (38.8%) are of the view government prioritize it, 60 (29.9%) disagree, and 63 (31.3%) are unsure. A strong relationship exists between women’s health prioritization and government’s commitment to cervical cancer prevention (*Phi (φc) = 0.666*). While 75 (37.3%) respondents were aware of a cervical cancer prevention and control policy, 124 (61.7%) were unaware. The relationship between women’s health prioritization and cervical cancer prevention and control policy is statistically significant (P*-value = .01*). Government commitment to prioritizing cervical cancer prevention is perceived as low among respondents. Only 24.4% of survey respondents think the government is committed, while a similarly low percent (31.3%) of respondents believe the government will introduce HPV vaccine. Respondents believe that women’s health is of low priority to the Ghanian government; only 38.8% find the government makes it a priority, reflecting the lack of confidence in the government to prioritize women’s health. Not surprisingly, 41.3% of respondents are of the view the government has the resources to implement national HPV vaccination. This low response communicates a lack of public confidence in government given its inaction on cervical cancer prevention.

### Right to Health

None of the sociodemographic factors (age, gender, highest education level, employment) had a statistically significant relationship to right to health (P*-value >.05)*. However, the relationship between women’s health prioritization and right to health was statistically significant (P*-value = .00*). Ninety-one (45.3%) respondents claim to have demanded their right to health at some point in their life in Ghana, while 53.7% have not done so. This may be indicative of a relatively passive population that are less likely to use social and/or political tools available to them to demand health as a fundamental human right. While this can cause governments to shift focus from health or reduce resource allocation to health, the constrained mobilization of interests inversely burdens society with preventable or curable diseases.

## Discussion

In elucidating stakeholders’ perspectives regarding the potential to develop and implement a nationwide HPV vaccination program in Ghana, key informants emphasized the need to enhance awareness, prioritize cancer prevention, and allocate resources for effective policy development and implementation. As the only existing policy document (NSCC) outlining a plan for cervical cancer control expired in 2016, no policy currently guides prevention and control strategies. This situation is not uncommon in Africa. For example, only 35 of 54 African countries have a policy on primary cervical cancer prevention, including HPV vaccination^49^ ^(p. 2)^. To influence policy decisions, it is imperative that advocacy groups elevate public awareness about cervical cancer and HPV vaccination and engage with key policymakers such as the GHS, the Ministry of Education (MoE), Civil Society Organizations (CSOs), and incumbent government. Notably, the Ghana Chapter of the International Papillomavirus Society (IPVS) is actively involved in nationwide awareness education.

In 2020, Ghana’s literacy rate (for those 15 years and above) was 80%,^50^ an increase from the 2020 (68%) and 2017 (65%) literacy rates for the sub-Saharan Africa region (excluding high income) (ibid). Awareness of HPV and mode of transmission is very high among educated people. A range of sources such as the internet, radio and online TV channels, and mobile phone platforms such as WhatsApp groups reflect channels for information dissemination. In a study of 288 Ghanaian women on HPV awareness, about 57% of educated women heard about HPV vaccine while less educated women had not^41^ ^(p. 899)^. In our study, about 68% female respondents had a university education, 77% heard about HPV, and 80% would take the vaccine if available; implying a link between education level, awareness, and vaccine acceptance. A systematic review on HPV vaccination noted that “inadequate community sensitization about HPV vaccine” was a constraint to awareness in Ghana^51^ ^(p. 711)^.

Awareness of HPV, HPV vaccination, and cervical cancer has been historically low in Ghana for the past decade. Healthcare workers,^37,38^ high school/college students,^52,53^ women,^41^ men,^54^ and mixed cohorts^55^ reflected low awareness levels. Limited awareness among health workers is concerning for population health surveillance, especially in less educated sub-populations with minimal resources.^56^ The absence of public education systematically removes sub-population groups with little formal education or sufficient capacity to decipher context heavy knowledge.

The actions or inactions of policy actors (which could activate structural modifiers such as a plan), expose multiple dimensions of sociostructural power dynamics wherein the more powerful and well-resourced prevail with undesirable health inequity. Public education enables the positioning of individuals’ health decisions. The drawback to this enablement is the detrimental action or inaction of dominant policy actors who possess significant resources to influence policy outcomes. Public education to promote population health knowledge remains a predominantly governmental decision guided by sociostructural underpinnings. The intersection of education and governmental action or inaction particularly within the realms of the “politics of prevention”^57,58^ is reflected in agenda-setting, manifested in reduced prioritization.^17,27^

In Ghana, prioritization of disease prevention and governmental resource allocation is dependent on how the disease is framed.^29^ ^(p. 55)^ While the government prioritizes technical training and guidelines, including the purchase of expensive mammography equipment for breast cancer for example, an absence of priority to purchase inexpensive equipment for cervical cancer screening persists, including tools for pap smear tests.^29^ ^(p. 54)^ The reason posited is that cervical cancer is perceived as a sexually transmitted disease brought about by risky sexual behavior.^59,60^ Alternatively, breast cancer receives tremendous local and international support with advocates pushing for governmental attention. Cervical cancer disproportionately affects women of poor socioeconomic status, unlike for breast cancer.^61,62^ This implies women with strong economic and political clout are more likely to be affected by breast cancer than cervical cancer. The networks supporting affluent women are more able to effectively frame, organize, and advocate for breast cancer intervention, in their interest.^29,63^ This reflects a “relational view of action”^64^ ^(p. 175)^ wherein actors leverage and use their resources, values, beliefs, norms, and position to create, frame or reframe a situation, and determine what has to be done.^65-67^ Mhazo and Maponga show that political elites can exert dominance in policymaking that evokes “self-interests at the expense of altruistic choices aimed at public benefit”.^63^ ^(p. 10)^ They offer a classic description of why interest in some public issues is politically organized by some interest group(s) and receives the needed political attention for policy reform, while others do not. Such organization of interest is what Schattschneider refers to as “mobilization of bias”.^68^ ^(p. 30)^ When public policy with public health benefits presents fiscal implications, they are more likely to be subjected to under-prioritization or receive pushback from actors/interest groups who will benefit less from the policy.^63^ ^(p. 5)^ However, when the benefits are high for powerful actors or elites who can manipulate the policymaking process, political will intensifies. Public policies receive traction based on the visibility, intensity, and the direction of the public problem.^68,69^ Perception of cervical cancer as a sexually transmitted disease (rather than a public health issue) dampens the visibility and required political intensity for policy action.

In Ghana, social mobilization for cervical cancer prevention (eg, HPV vaccination) is low or nearly non-existent.^51^ ^(p. 711)^ Even with the under-prioritization of women’s health in general, among diseases of nearly equal mortality and morbidity, there is inequitable health resource allocation. The inequity extends government’s inclination to drift towards constructs that appeal to a larger and a strongly knit group with common interests. Consequentially, this inequity stigmatizes one disease over another with the same spectrum of mortality and morbidity. When policymakers lose sight of a public problem due to competing interests, it reflects policy inertia and a low level of accountability to the people. Those unable to effectively mobilize resources and are allocated minimal political power to influence policy by virtue of their socioeconomic and political positions, tolerate inequitable policy or reforms. This undermines the right to health and can result in lax accountability, especially from government. Such a prevailing situation, reflected by respondents (53.7%) who have not demanded a right to health, reinforces a norm while it entrenches patterns of government inaction in agenda setting for disease prevention. Baum and colleagues summarize that for public policies or reforms which present the “the greatest potential to reduce inequalities, they generate the least political will as they threaten those benefiting from the status quo”.^70^ ^(p. 3)^

While the government remains the central actor in public policymaking, pressure to intensify the visibility of a public issue can equally come from organized interest groups who can garner political will.^63,70,71^ This implies actors such as the MOH and GHS must be studied or evaluated beyond their nuanced characteristics (eg, beliefs and values) with a focus on their collective behavior in the network. Understanding the behavior of policy actors is important in the policymaking process, however, from an actor network perspective, it should not be the “focal point,” rather, the “connection between them [ie, policy actors] through which they act”.^72^ ^(p. 175)^ Thus, politics and its overlay with power to manipulate behaviors, control available options, and formulate policies for society, advertently disadvantages some groups while increasing the advantages for other groups which perpetuates “systematic inequality [that] flow[s] from membership in one class rather than another”.^73^ ^(p. 377)^ In essence, actions or inactions of governments or interest groups become policies that practically block opportunities to quality health. The policy actors with the ability to allocate power and confer political will directly create inequity in health as those who are unaware of and/or cannot afford the HPV vaccine become further disenfranchised. A lack of mobilization of resources by policy actors defines influence and how power is allocated, which can block opportunities that yield beneficial outcomes contributing to health inequity.^72^ ^(p. 82)^ Rwanda’s pioneering nationwide HPV vaccination program in Africa in 2011 illustrates that with unwavering political commitment to health, even in the face of limited resources, governments can mobilize support from relevant stakeholders to successfully achieve their health goals.^7^

### Policy Implications

To advance Gavi’s commitment to accelerating HPV vaccination and averting preventable cervical cancer deaths, it is imperative that governments of Gavi-eligible countries collaborate with relevant stakeholders to establish and sustain national HPV vaccination programs, allocating resources to fortify weak health systems. Ghana has well-established policy and programmatic responses for non-communicable diseases (NCDs), but evidence reveals a lack of practical attention and political will to address cervical cancer despite its cost-effectiveness.^74-77^ The lack of political will is reflected in the low number of respondents (12.9%) who find the government’s effort to educate the public on HPV is adequate as opposed to 71.1% respondents who believe the government is not doing enough. Focusing on the behavior of policy actors, the “recursive interactions” within the networks and the evolution of networks implies attention to the interactions taking place and the outcomes they generate.^64^ ^(p. 175)^ The analysis identifies key areas of concern and emphasizes the urgent need for concerted efforts to implement nationwide HPV vaccination as an essential strategy in the prevention of cervical cancer. A notable finding is that a small number of study respondents reported initiating sexual activity before the age of 17, underscoring the critical importance of prioritizing HPV vaccination for school-aged children and adolescents in Ghana.

### Future Work

Characterizing the various actors within Ghana’s policy networks who shape cervical cancer prevention or health-promoting programs would further support analysis of cervical cancer prevention policy in Ghana. Systematically mapping out the policy actors and the process through which policies are developed - including the representation of various stakeholder groups in policy prioritization - would enhance understanding of the policymaking process. Such analysis may reveal potential levers for policy change, particularly by highlighting opportunities to strengthen the inclusion and influence of relevant stakeholders.

### Strengths and Limitations

The study includes input from a range of relevant experts and a general survey of individuals with diverse backgrounds including in academia, women’s advocacy organizations, physicians, media, HPV researchers, and a section of the general public. This diversity provided comprehensive perspectives for context and understanding of the delay in nationwide HPV vaccine program. Limitations of the study are that key informant interviews were conducted from 05 January 2022 to 05 April 2022, and survey responses were received from 21 December 2021 to 15 March 2022. The limited timespan to collect data may have impacted opportunities to interview more stakeholders, even though 8 key informants provide thematic stability. The mixed methods approach that involves triangulation of data sources compensate for this shortcoming. Together, the samples are statistically sufficient and provide both depth (qualitative saturation) and breadth (quantitative precision) and aligned with established methodological standards for HPV vaccination implementation research.

## Conclusion

In assessing a context of high incidence of cervical cancer, coupled with low public disease awareness and low governmental priority for cervical cancer prevention in Ghana, key informant perspectives shed light on these challenges and how governance processes could potentially alleviate them. The analysis found that awareness of HPV, the HPV vaccine, and cervical cancer, though still low, is gaining momentum, in contrast to previous studies that reflected persistently low awareness, this is expected to gradually increase particularly among literate populations, as the less literate catch up. The increased awareness may support policy entrepreneurs and societal groups to advocate for prioritization of cervical cancer prevention and control. The survey findings show a high level of vaccine confidence among respondents, suggesting that HPV vaccine uptake in Ghana promises to be high once a nationwide HPV vaccine program is implemented. Given the health intervention reports, draft policies, and health insurance schemes in place, Ghana is not deficient in policy or program design. A fundamental policy issue the study highlights is that policy implementation in Ghana is inhibited by low priority setting and inadequate resource allocation. The support Gavi offers for HPV vaccine programs, that the Ghanaian government may leverage creates a window of opportunity; especially as the costs of an HPV vaccine program are lower than those of not offering a program given its preventive effects.

## Supplemental Material

Supplemental Material - Challenges and Opportunities for Cervical Cancer Prevention Through HPV Vaccination in Ghana: A Public Health Policy Analysis Supplemental Material for Challenges and Opportunities for Cervical Cancer Prevention Through HPV Vaccination in Ghana: A Public Health Policy Analysis by Eric Asempah, Ene Ikpeb, Michelle Wyndham-West, Mary E. Wiktorowicz in Cancer Control

Supplemental Material - Challenges and Opportunities for Cervical Cancer Prevention Through HPV Vaccination in Ghana: A Public Health Policy Analysis Supplemental Material for Challenges and Opportunities for Cervical Cancer Prevention Through HPV Vaccination in Ghana: A Public Health Policy Analysis by Eric Asempah, Ene Ikpeb, Michelle Wyndham-West, Mary E. Wiktorowicz in Cancer Control

Supplemental Material - Challenges and Opportunities for Cervical Cancer Prevention Through HPV Vaccination in Ghana: A Public Health Policy Analysis Supplemental Material for Challenges and Opportunities for Cervical Cancer Prevention Through HPV Vaccination in Ghana: A Public Health Policy Analysis by Eric Asempah, Ene Ikpeb, Michelle Wyndham-West, Mary E. Wiktorowicz in Cancer Control

## Data Availability

The data underlying this article will be shared on reasonable request to the corresponding author.*
