# Supplementary material for: Challenges and Opportunities for Cervical Cancer Prevention Through HPV Vaccination in Ghana: A Public Health Policy Analysis
Source: Cancer Control. 2025 Oct 3;32:10732748251383280. doi: 10.1177/10732748251383280 (PMC12495208; doi:10.1177/10732748251383280)
Supplement: Supplemental Material - Challenges and Opportunities for Cervical Cancer Prevention Through HPV Vaccination in Ghana: A Public Health Policy Analysis [file sj-pdf-1-ccx-10.1177_10732748251383280.pdf]

Supplementary File 1

Interview Guide

DURATION IN MINUTES	RESEARCH INTERVIEWING TASK		
**15**	**INTRODUCTION (Housekeeping)**		**RATIONALE (To)**
1	✓ Greet and thank participant for her/his time		1. Create Rapport with Interviewee and establish connection during the interviewing process.<br>2. Ensure all documents are signed and the interviewee understands the objective of the interview.<br>3. Collect signed inform consent form prior to starting the interviewing process.
2	✓ Disseminate Research Consent form and explain it to the participant		
1	✓ Welcome Participant and Introduce self to participant		
1	✓ Allow Participant to Introduce herself or himself		
1	✓ State the Research Problem to the Participant		
2	✓ State the Research Objectives to Participant		
3	✓ Explain to Participant the Interview Process		
2	✓ Explain to Participant the Ethical Protocols of the Research		
2	✓ Explain to Participant their rights during the interview process and Assurance that their information will be held in high Confidentiality		
**50**	**INTERVIEW QUESTION**	**PROBE**	**RATIONALE (To understand)**
8	What is your general view on HPV associated cervical cancer in your country?	Do you think people have been educated enough on HPV associated Cervical cancer?	General Perspective in HPV Associated Cervical Cancer
8	In your opinion, is cervical cancer given adequate governmental attention/priority? Could you kindly provide some explanation for your answer?	Should HPV Vaccination for adolescent (for example) be a priority for the government? Can you kindly explain your position/answer?	Governmental priority and agenda settings for HPV vaccine uptake and policy/program
10	Can you describe the public health policymaking approach in your country?	Is health equity built in the policymaking process?	Health policy points of convergence and/or divergence
8	Who are the key players or stakeholders in vaccine policymaking process in your country?	Is the media influential in vaccination uptake in your country? If so, which means are used and which ones have been effective in your opinion, and why you think so?	Core actors inside and outside of government
8	What process is normally followed to introduce a new vaccine or new therapy for coverage? Does a particular committee review such proposals? How are priorities determined?	What strategy (if any) does the government utilize in vaccine purchasing negotiation? Example: negotiation with vaccine manufacturer, negotiate through GAVI, negotiate through other stakeholders, etc.<br>How transparent is vaccine negotiation in your country? What is the general view of the population to be vaccinated?	Actor's level of influence in HPV vaccine access, Availability, and Population Acceptability of Vaccine
8	Can you explain how citizens in your country demand right to health from the government or have done so in the past?	Are human rights ideals prominent in Public Health in your country?	Governments Actions leading to health equity or inequity
