# Supplementary material for: Challenges and Opportunities for Cervical Cancer Prevention Through HPV Vaccination in Ghana: A Public Health Policy Analysis
Source: Cancer Control. 2025 Oct 3;32:10732748251383280. doi: 10.1177/10732748251383280 (PMC12495208; doi:10.1177/10732748251383280)
Supplement: Supplemental Material - Challenges and Opportunities for Cervical Cancer Prevention Through HPV Vaccination in Ghana: A Public Health Policy Analysis [file sj-pdf-2-ccx-10.1177_10732748251383280.pdf]

NVivo Themes generation[1]

Code Names	Description	Files	References
**AWARENESS**	**Participants Awareness of HPV and HPV Related cervical cancer**	**5**	**10**
Low Awareness	Awareness Level	1	1
Cervical Cancer	Knowledge of Cervical Cancer and Relation to HPV	2	3
Training	Training for Healthcare Professionals	1	2
Direction for prevention	Approach to preventing HPV-Related Cervical Cancer	1	4
HPV Infections	HPV Infections and Cervical Cancer Prevalence	1	1
Education	Public Education on HPV-Related Cervical Cancer	3	3
National Vaccination Program	Plans towards National HPV Vaccination program	2	9
**Media Influence in Vaccine uptake**	**Media Influence in Vaccine Uptake in Ghana**	**7**	**11**
Prevention	Prevention of cervical cancer in Ghana	5	6
Screening	Women screening for cervical cancer	3	5
Vaccination	Vaccination regime in Ghana	3	8
Vaccine Dose	HPV doses	1	2
Vaccines	HPV Vaccines availability	1	3
**POLICYMAKING**	**Policymaking process towards cervical cancer prevention and control**	**3**	**9**
Policy	Policy in place to prevent and control cervical cancer	5	6
Prevalence	Cervical cancer prevalence in Ghana	2	7
Screening	Women screening for cervical cancer	3	5
Prevention	Prevention of cervical cancer in Ghana	5	6
Primary Prevention	Primary prevention of cervical cancer	1	2
Secondary Prevention	Available secondary prevention against cervical cancer	1	1
Stakeholders' vaccine policy	What vaccine policy is in place	5	9
**PRIORITY SETTING**	**How dedicated is the government to prevent cervical cancer**	**7**	**16**
Negative outlook	How is cervical cancer perceived	2	2
Vaccines	HPV vaccine availability	1	3
Vaccination Prioritization	Core vaccine priority areas	2	3
vaccine purchase negotiation	Vaccine purchase negotiation strategies	2	4
Vaccine comparative pricing	Vaccine procurement strategies	1	1
Women's health prioritization	Prioritization of women's health in Ghana	4	6
Children's Health prioritize over women	Comparing Women's Health priority to Children	1	1
**RESOURCE ALLOCATION**	**How much of healthcare resource is allocated to cervical cancer**	**4**	**7**
Donors	Donors towards HPV-Related Cervical Cancer Prevention	5	8
Funding	Funding Potentials towards HPV-Related Cervical Cancer	2	4
Healthcare Expenditure	Resource Allocation and Government Priority Setting	3	3
**RIGHT TO HEALTH**	**How the public demand right to health**	**3**	**7**
Health Equity	Health Equity among gender lines	1	1

[1] *Themes generated are in bold.*
