# Supplementary material for: Challenges and Opportunities for Cervical Cancer Prevention Through HPV Vaccination in Ghana: A Public Health Policy Analysis
Source: Cancer Control. 2025 Oct 3;32:10732748251383280. doi: 10.1177/10732748251383280 (PMC12495208; doi:10.1177/10732748251383280)
Supplement: Supplemental Material - Challenges and Opportunities for Cervical Cancer Prevention Through HPV Vaccination in Ghana: A Public Health Policy Analysis [file sj-pdf-3-ccx-10.1177_10732748251383280.pdf]

Supplementary File 3

Survey associations

Cross Tabulation	*Phi Coefficient(φc)*	*P-Value*
Highest Education Level * Government commitment to cervical cancer prevention	0.303	0.190
Highest Education Level * Awareness of cervical cancer prevention	0.389	0.000
Highest Education Level * Awareness of HPV Vaccine	0.368	0.010
Highest Education Level * Willingness to be Vaccinated	0.251	0.178
Highest Education Level * Policy on cervical cancer Prevention and Control	0.170	0.219
Highest Education Level * Government Resources for HPV vaccination Control	0.209	0.360
Highest Education Level * Right to health Control	0.117	0.606
Age of debut sex * Aware of HPV related cervical cancer	2.710	0.070
Gender * Medium of HPV awareness cancer	0.284	0.640
Gender * Willingness to be vaccinated	0.379	0.000
Government Commitment to Cervical cancer prevention * Public Education on HPV related cervical cancer	0.515	0.000
Women health prioritization * Government commitment to cervical cancer prevention	0.666	0.000
Women health prioritization * Policy on cervical cancer prevention and control	0.272	0.001
Gender * Right to health cancer	-0.006	0.935
Age range * Right to health cancer	0.360	0.878
Highest education level * Right to health cancer	0.117	0.606
Employment * Right to health cancer	0.220	0.870
Age of debut sex * Right to health cancer	0.161	0.278
Women health prioritization * Right to health	0.301	0.000

SPSS descriptive analysis
